# Notch-free vermillion after unilateral cleft lip repair: The Charles Pinto centre protocol

**DOI:** 10.4103/0970-0358.44935

**Published:** 2008

**Authors:** P. V. Narayanan, H. S. Adenwalla

**Affiliations:** Department of Plastic Surgery, Burns, Charles Pinto Centre for Cleft Lip, Palate and Craniofacial Anomalies, Jubilee Mission Medical College and Research Institute, Trichur-680 005, Kerala, India

**Keywords:** Primary cleft lip repair, vermillion notch, Z plasty

## Abstract

A notch on the vermillion is one of the most common complications following the repair of a unilateral cleft lip. Several methods have been described for the secondary correction of a notch. However, there are only a few reports on how the notch can be prevented during primary lip repair. Causes of a vermillion notch were analysed at the Charles Pinto Centre for Cleft Lip and Palate and each possible cause addressed by an appropriate procedure. This protocol was then followed in every patient. In this manner, we have been able to avoid notches in unilateral cleft lips altogether and more significantly, junior trainees in our department have also been able to consistently avoid a notch in their repairs.

## INTRODUCTION

A notch is a common blemish following the repair of a unilateral cleft lip. The term “notch” is used to denote a break in the free border of the vermillion. Although the notch has been recognised as one of the most common complications after unilateral cleft lip repair,[1] a survey of published literature does not reveal many publications of methods to primarily avoid a notch.

The senior surgeon at our centre, Dr. H. S. Adenwalla, analysed the main causes leading to the formation of a notch and addressed them using a protocol which has been adhered to on all unilateral cleft lip patients operated on at our centre. As a result, we have been able to consistently obtain a notch-free vermillion.

### Causes of vermillion notching:

- Inadequate rotation of the medial element of the lip resulting in a tented-up Cupid's bow point on the cleft side and a notch on the vermillion
- Turning-in of the sutured edges
- Deficiency of bulk of the *Orbicularis Or is* at the vermillion
- Contracture of the straight line scar on the mucosal aspect of the lip

### The Charles Pinto Centre Protocol:

- Having noted the above causes, an attempt was made to correct each of them. We use the Millard's Rotation Advancement technique in all our unilateral cleft lips.
- Adequate rotation of the medial element with an ample back-cut in all patients [Figures 1a and b].

**Figure 1(a):**
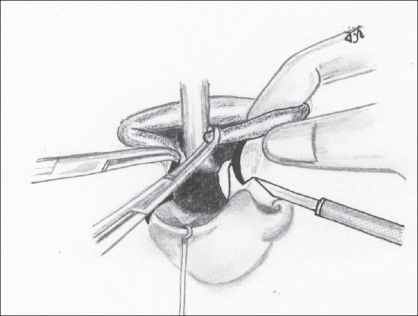
The back-cut about to be performed perpendicular to the rotation incision

**Figure 1(b):**
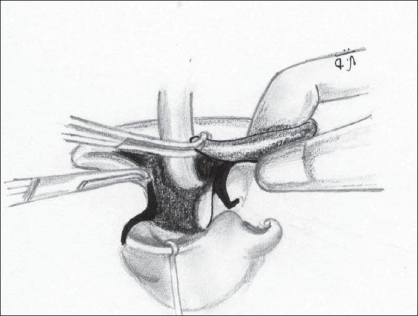
Back-cut completed; Cupid's bow peak points are at the same horizontal level
- Undermining the skin and mucosal edges to prevent their turning in. This undermining is limited to a few millimetres from the cleft edges [Figure 2].

**Figure 2:**
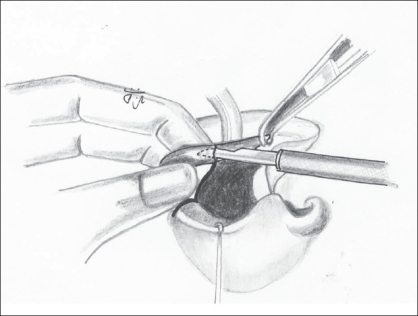
Undermining the vermillion on the cleft side; the noncleft side vermillion is also similarly undermined
- While paring the vermillion, an excess of muscle tissue is retained on both the medial and lateral elements. As a result, there is a good bulk of muscle tissue that acts as a filler [Figure 3]. At least three 6-0 Ethilon sutures are placed to bring this muscle together, thus creating the appearance of “a roll” or “sausage” [Figure 4].

**Figure 3:**
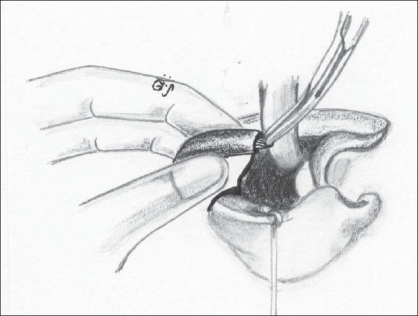
Shows the excess of orbicularis oris left behind while paring the vermillion

**Figure 4:**
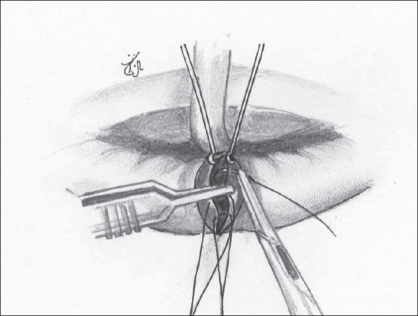
The muscle left behind on both the sides being sutured with 6-0 ethilon; a minimum of three stitches
- To counteract the straight line scar contracture, a Z plasty is mounted on the mucosal aspect of the lip [Figure 5].
- All the above mentioned steps are done in all our unilateral cleft lip patients. The long-term results of patients operated in this manner are shown in Figures 6a, b and 7a, b.

**Figure 5:**
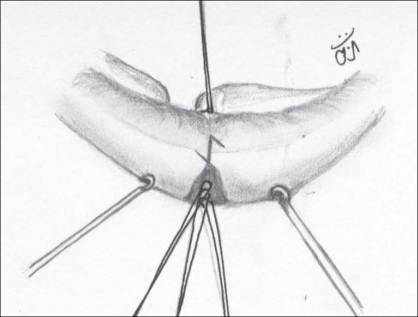
Z plasty on the mucosa

**Figure 6a:**
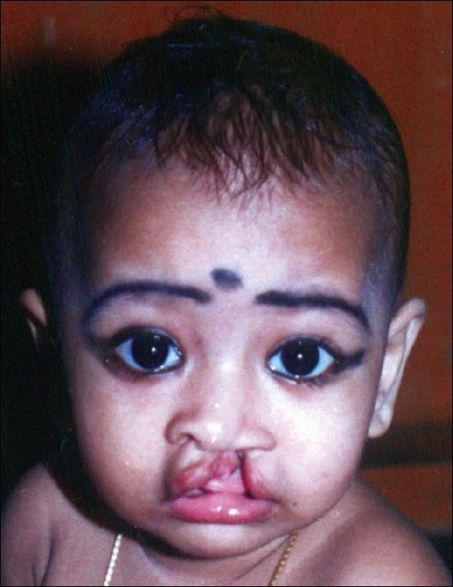
Preoperative photo

**Figure 6b:**
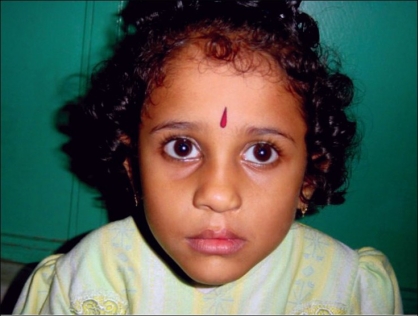
Five years postoperative photo

**Figure 7a:**
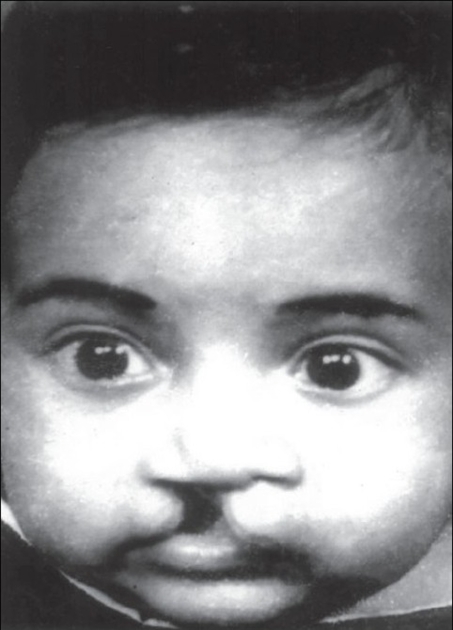
Preoperative photo

**Figure 7b:**
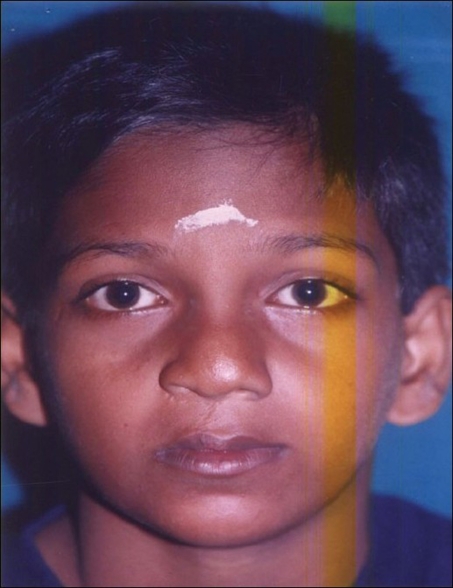
Eleven years postoperative photo

## DISCUSSION

A review of literature reveals that although a vermillion notch has been recognised as one of the most common complications following unilateral cleft lip repair,[1] publications on the method of prevention of such notches are hard to come by. Noordhoff[2] has advocated the use of a triangular vermillion flap devised from the redundant vermillion on the cleft side to compensate for the deficiency of vermillion on the noncleft side. This method is logical and popular. However, it requires great finesse to execute and often fails in the hands of the younger less experienced cleft surgeon.

The Charles Pinto Centre Protocol is taught to many trainees, who have then proceeded to perform numerous lip repairs without any notching. We have followed up our patients for over 15 years and have consistently avoided a notch.

The rotation with a back-cut should be adequate to align the Cupid's bow points in the same horizontal plane. Care must be taken not to cross the philtral column on the noncleft side as this would cause lengthening of the noncleft side as well.[3] In the early postoperative period, the Cupid's bow on the cleft side may appear to be pulled up, resulting in a notch on the vermillion. However, if the original rotation has been adequate, this Cupid's bow will settle down in the ensuing months; the notch then naturally disappears.

Undermining of the skin and mucosa is limited to a few millimetres from the edges; excessive undermining itself may lead to a curling-in of the edges.

It is important to preserve a good bulk of muscle on the vermillion while paring. While this may be difficult to start with, one soon manages to retain a good amount of tissue with practice. It is possible to overdo this, leaving a bulge in the vermillion, which if very obvious, has to be thinned later on. The muscle bulk on the noncleft side is always less than its counterpart on the cleft element. One has to be extremely patient in paring the noncleft side vermillion.

If the Z plasty on the mucosa is placed too far anteriorly, it shows and if it is placed too far back, it does not serve its purpose. The correct placing of the Z is learnt with experience, and must bring Noordhoff's red line in alignment. The method is simple and reproducible.

## CONCLUSION

We have described the protocol followed at our centre to prevent the occurrence of a notch on the vermillion after unilateral cleft lip repair. It is a simple logical sequence of steps that are easy to follow, with reproducible results.
